# Prevalence of Sero-Molecular Markers of Hepatitis C and B Viruses among Patients with *β*-Thalassemia Major in Northern West Bank, Palestine

**DOI:** 10.1155/2018/1039423

**Published:** 2018-09-05

**Authors:** Kamal Dumaidi, Amer Al-Jawabreh, Fekri Samarah, Maha Rabayaa

**Affiliations:** ^1^Department of Medical Lab Sciences, Arab American University, Palestine, State of Palestine; ^2^Al-Quds Public Health Society, Jerusalem, State of Palestine

## Abstract

**Background:**

HCV and HBV present a great challenge in the management of* β*-thalassemia patients.

**Objective:**

The present study aimed to determine the prevalence of both HBV and HCV in multitransfused-dependent *β*-thalassemia patients in northern West Bank, Palestine, using sero-molecular markers.

**Methods:**

Serum sample from 139 multitransfused *β*-thalassemia patients were tested for HBV and HCV markers including HBsAg, anti-HBc, anti-HBs, HBV-DNA, and anti-HCV and HCV-RNA. Demographic data and selected clinical parameters were collected by means of a questionnaire and from the patients' medical files.

**Results and Conclusion:**

The mean (±SD) age of patients was 18.1 years (±10.6). The overall prevalence of the HCV was 10% (14/139), which is 50 times higher than the normal Palestinian population (0.2%). Of which, 3 were positive for anti-HCV alone, 7 positives for HCV-RNA alone, and 4 positives for both anti-HCV and PCR-RNA. On the other hand, low prevalence of HBV was detected at a level of 0.7% (1/139). Only one patient had HCV-HBV coinfection. Twenty-five patients (19%) were positive for anti-HBc, while 99 (71%) were immune with the anti-HBs level above 10 IU/mL. Anti-HBc was insignificantly high (*P*=0.07) in HCV-positive cases. In conclusion, the prevalence of HCV among *β*-thalassemia patients is considered high compared to normal population. Determination of HCV prevalence should be based on the detection of both HCV-RNA and anti-HCV. On the contrary, HBV showed a low prevalence. A follow-up schedule and administration of booster dose of HBV vaccine is strongly recommended for *β*-thalassemia patients whose anti-HBs level <10 IU/ml.

## 1. Background

Thalassemia is a group of hereditary diseases characterized by hemolysis of red blood cells due to defect in *β*-globin chain synthesis, which leads to a decreased synthesis of *β*^+^ or complete absence *β*^o^ of the *β*-chains. *β*-Thalassemia is classified clinically according to disease severity into three major subtypes: the asymptomatic *β*-thalassemia trait (BTT), moderate *β*-thalassemia intermediate (BTI), and the severe form or transfusion dependent *β*-thalassemia major (BTM). The disease can also be in combination with other hemoglobinopathies such as *β*^S^ or *β*^C^ [1, 2]. Approximately 1.5% (1–20%) of the world population is known to be carriers for *β*-thalassemia. High prevalence of the *β*-thalassemia carrier was reported in the Mediterranean region, Africa, Southeast Asia, and the Middle East [1, 3, 4]. Management of thalassemia patients depends mainly on regular blood transfusions; however, complications including, iron overload and transfusion-transmitted infections were reported, which may therefore increase the rate of morbidity and mortality [1, 5]. *β*-thalassemia patients are at high risk of acquiring viral infections such as hepatitis B virus (HBV) and hepatitis C virus (HCV). Although, the incidence of viral hepatitis among thalassemia patients has been reduced following the implementation of HBV vaccine and the screening of transfused blood components for HBV and HCV, significant prevalence of HBV and HCV among thalassemia patients are still reported [6]. Previous studies show a substantial prevalence of HBV and HCV among *β*-thalassemia patients in Egypt, Pakistan, and Iran ranging from 0 to 4.12% for HBV and 6.8 to 82% for HCV [7–18].

In Palestine (the West bank and Gaza Strip), *β*-thalassemia is still considered a major public health problem, despite the obligatory premarital screening policy, where 895 (575 in the West bank and 320 in Gaza Strip) thalassemia patients are currently treated with transfusion and chelating agents [19]. HBV and HCV along with HIV and syphilis are adopted as routine tests for blood units in blood banks in Palestine [20]. In the prevaccine era, Palestine had been classified as an area of high endemicity for hepatitis B (carrier rates range from 2 to 18.5%) [21]. Therefore, in 1992, the Palestinian Ministry of Health implemented a national obligatory hepatitis B vaccination program for newborns which was expanded in 1994 to cover all household contacts of HBV carriers and other high-risk groups including health care workers, patients with multiple blood transfusions such as thalassemia patients and other risk groups. Accordingly, reduction in the HBV infection rate was reported with incidence rate of 23/100,000 and 0.07/100,000 for HBV and HCV, respectively, in the West Bank, Palestine [20]. To the best of our knowledge, no previous study has investigated the status of HBV and HCV in *β*-thalassemia patients in the West Bank, Palestine. Therefore, the aim of the present study was to describe the prevalence of both HBV and HCV among *β*-thalassemia patients in the northern part of the West bank, Palestine, using serology and molecular assays.

## 2. Methods

### 2.1. Study Population

In this cross-sectional study, 139 *β*-thalassemia patients attending the thalassemia units in three major hospitals in the West Bank, Jenin government hospital in Jenin, Al-Watani government hospital in Nablus—the most populous city in northern West Bank, and Tulkarem government hospital in Tulkarem during the period from June 2014 to September 2015, were investigated for HCV and HBV infections. Inclusion criteria included confirmed cases of *β*-thalassemia patients regularly receiving at least one blood unit monthly with last transfusion four weeks prior to sampling. The patients' medical files were reviewed for demographic data, clinical history, and laboratory test results. Patients were interviewed to double check the data. Demographic data included age, sex, and place of residence, while clinical history included the type of thalassemia, date of diagnosis and first blood transfusion, frequency of blood transfusion, history of HBV vaccine, and overt HBV and HCV. A written informed consent was obtained from each participant or parents/guardians, in the case of children. The study was also approved by the Palestinian Ministry of Health in West Bank, Palestine, under the reference number 162/1075/2014.

### 2.2. Laboratory Investigations

Five milliliter of venous blood sample was collected in a sterile plain tube from each *β*-thalassemia patient. After clot, the tube was centrifuged for 10 minutes at 3000 rpm. Then, the serum was separated into two sterile 1.5 ml microtubes and stored at −20°C until use. Anti-HCV and HBsAg were detected using a fourth-generation ELISA which is based on chemiluminescent microparticle immunoassay (CMIA) for the qualitative detection of anti-HCV and HBsAg in serum or plasma sample (Architect-I1000SR-Abbott, Santa Clara, California, United States of America). Determination of anti-HBs and anti-HBc was carried out using the commercially available EIA kits (ELISA; Human Gesellschaft FuerBiochemica und Diagnostica, Wiesbaden, Germany) according to manufacturer's instructions. Samples were run in duplicates. Samples showing anti-HBs titer more than 10 IU/mL were considered protective according to Jack et al. [22].

### 2.3. Molecular Assay

Viral HBV-DNA and HCV-RNA were extracted from 200 *μ*l of serum using the QIAamp viral RNA/DNA extraction kit according to the manufacturer's instructions (Qiagen, Hilden, Germany). For HBV, the viral DNA was amplified using nested PCR with two primer sets targeting the viral polymerase gene described previously by Selabe et al. [23]. For HCV, the viral RNA was also amplified using reverse transcriptase nested PCR with two primer sets targeting a partial part of the 5' NCR described previously by El-Kader et al. [24]. PCR ReddyMix reagents were used (Thermo Fisher Scientific Inc.). The appearance of a 647 bp and 235 bp bands was considered positive for HBV and HCV, respectively. Negative control using nuclease-free water and a positive control were run.

### 2.4. Statistical Analysis

The Epi Info™ 7 statistical package (Centers for Disease Control and Prevention, Atlanta, USA) was used for data management and analysis. Fisher's exact test and chi-squared test were used to establish an association between variables. Frequency tables and percentages were calculated. The statistical difference was considered significant when *P* < 0.05.

## 3. Results

A total of 139 *β*-thalassemia patients participated in the study. Serum samples were collected from the patients attending the three hospitals. One hundred and nineteen of the patients (85.6%) had been diagnosed as major or intermediate *β*-thalassemia, 15 (10.9%) as sickle *β*-thalassemia, and 5 (3.5%) as sickle cell anemia. The mean (±SD) age of patients was 18.1 (±10.6) years with a median (50% percentile) of 16 years and 75% percentile of 24 years. In the study group, males were insignificantly higher than females (*P*=0.79). All of the 139 patients were vaccinated for HBV (110 were vaccinated at birth and 29 vaccinated in 1994 during treatment) (Table 1).

A total of 14 thalassemia patients (10%) were found to be positive for HCV, of which 3 were positive for anti-HCV alone, 7 positives for HCV-RNA alone, and 4 positives for both. The general characteristic and the laboratory profile of the HCV-positive cases are shown in Table 2. There is only one (0.7) patient found to be positive for HBsAg by serology, but none were positive for HBV-DNA by PCR. One patient had HBV-HCV coinfection by serology but negative by molecular assays for both viruses. Twenty-five patients (19%) were positive for anti-HBc, while 99 (71%) were immune with anti-HBs level above 10 IU/ml. The level of immunity with anti-HBs≥ 10 IU/ml in anti-HBc positive and negative patients was found to be statistically indifferent (*P*=0.63). The mean hemoglobin level was below normal (normal range ≥9.5 g/dl) as the study group consisted of thalassemia patients. The clinical and laboratory parameters of the study sample are shown in (Table 3).

By comparing clinical and laboratory data of the HCV-positive thalassemia patients with HCV-negative patients of the same group, it was found that jaundice and AST levels were the only parameters that showed significant differences between the two groups. In addition, anti-HBc was higher in HCV-positive patients, but not significantly (*P*=0.07). Thirteen of the HCV-positive cases (93%) were above 12 years old, indicating that prevalence of HCV among the patients were slightly elevated at higher age, but not significantly (*P*=0.06). Clinical and laboratory parameters of both HCV-positive and HCV-negative patients are shown in Table 4.

## 4. Discussion

The prevalence of the HCV among thalassemia patients in Palestine was 10%. Out of which, half were positive for HCV-RNA, one-fifth were positive for anti-HCV, and one-third were positive by both. The history of jaundice was the main clinical feature in HCV thalassemia patients. On the other hand, the prevalence of HBV was negligible. The prevalence of HCV among *β*-thalassemia patients as revealed by this study is 50 times higher than in normal Palestinian population, 10% compared to 0.2%, with similar folds in the neighboring countries such as Jordan (0.3%), Lebanon (0.2%), and Syria (0.4) [25]. The high prevalence of HCV among thalassemia patients could be attributed to the transmission of anti-HCV negative components during the serologically negative window period. Also, it could be due to occult HCV which is the presence of undetectable levels of genomic HCV-RNA strands in liver biopsy or peripheral blood mononuclear cells (PBMC) with the absence of anti-HCV antibodies in the plasma by conventional laboratory methods [26, 27]. In addition, HCV nosocomial infection among thalassemia patients had been reported [28]. A consistent prevalence of HCV among thalassemia patients had been reported in Jordan (16.3%) and Iran (6.8%) [8, 29]. On the contrary, higher prevalence rate among frequently transfused *β*-thalassemia patients had been reported in endemic countries such as Egypt and Pakistan [7, 9].

The present study showed that 13/14 (93%) of HCV-positive cases are older than 12 years, but not significant (*p* = 0.06). Atwa et al. reported that age is an important predictor for HCV infection among thalassemia and nonthalassemia patients in endemic areas [11, 30]. In the present study, 35.7% of the HCV-infected patients were positive for anti-HBc antibodies which was found to be higher among HCV-infected thalassemia patients, but not significant (*P*=0.07). This is in agreement with the study of Arababadi et al. which reported that 33% of HCV-positive patients had anti-HBc antibodies. Furthermore, Shaker et al. reported association between HCV infection and the presence of anti-HBc, the indicator to previous HBV exposure, among thalassemia patients [31, 32]. In general, the presence of anti-HBc in HBV-negative patients regardless of their HCV status is an indicator of previous HBV exposure and solid vaccine protection against HBV. The significant increase in AST levels and the reported history of jaundice in HCV-negative cases indicate that thalassemia patients may have suffered from hemolysis and/or transient deterioration of the liver function irrespective of any apparent viral infection.

The determination of HCV prevalence in general population as well as in thalassemia patients is so far based mainly on the detection of anti-HCV antibodies using the ELISA test with PCR for confirming positive cases as a two-stage (sequential) testing strategy. Although serological assays are reliable and accessible with relatively low cost in diagnosing HCV infection, they may fail to diagnose infection during the window period as well as in immunocompromised patients. In this study, the prevalence of HCV estimated at 10% was determined by detecting both anti-HCV antibodies and HCV-RNA. Seven of the 139 samples (5.03%) were seronegative for anti-HCV, but found to be positive for HCV-RNA (HCV viremia). Similar instances of HCV viremia had been reported from seronegative serum samples in blood donors (0.66%), hemodialysis patients (10%), and HIV patients (38.2%) [33–35]. The discrepancy in the prevalence of HCV viremia from the seronegative samples between our study and the others could be due to rate of HCV endemicity, study population, and the sensitivity of the molecular assay used. This case indicated that using the simultaneous two-stage strategy improved the detection rate of HCV viremia. In addition, the implementation of a highly sensitive molecular assay for HCV diagnosis is an added value for the early diagnosis of HCV from seronegative patients which is decisive in treatment regimen, taking into consideration, the HCV viral load, genotype, and the clinical status of the patients [36, 37].

On the other hand, this study showed that HBV infection remained low (0.7%, 1/139) relative to the normal population (1.85%), indicating effectiveness of the immunization policy to vaccinate newborns and the thalassemia patients [20]. The low prevalence of HBV among *β*-thalassemia patients in Palestine was consistent with results reported in Iran (1.1%) and Malaysia (1%) [14, 38, 39]. However, higher prevalence had been reported in endemic area including Egypt (3–29%) and Pakistan (3%) [11, 40, 41]. Furthermore, 25 (19%) of our cases had anti-HBc with all having negative results for both HBsAg and HBV-DNA by PCR. In addition, our study showed that most of the *β*-thalassemia patients (71%) still have immunity against HBV infection (anti-HBs ≥ 10 IU/ml) which is high and comparable to healthy individuals as had been reported earlier by our group [42] and others [43, 44]. Azarkeivan et al. showed that the immunoprotection rate of 416 thalassemia patients against HBV increased from 46.9 up to 69.4% after receiving a booster dose for those who had anti-HBs ≤10 IU/ml [45]. Therefore, the decline in the HBV infection rate among thalassemia patients and the simultaneous absence of HBsAg and HBV-DNA in anti-HBc positive cases in our study reflect vaccination protection and wide coverage, effective screening of the blood donor, and the improvement in the public perception towards HBV infection during the last decade.

In conclusion, the prevalence of HCV among *β*-thalassemia patients is significantly higher than the normal population. It is recommended to determine HCV infection in thalassemia patients based on the detection of HCV-RNA by PCR and anti-HCV by serology, simultaneously. On the contrary, the study showed low prevalence of HBV. Finally, a follow-up schedule and administration of booster dose of HBV vaccine is strongly recommended for thalassemia patients whose anti-HBs level <10 IU/mL.

## Figures and Tables

**Table 1:** Demographic characteristics of the study group (*N*=139).

Parameter	*N*	%
Sex		
Male	71	51
Female	68	49
Age group		
0–4	10	7
5–9	16	12
10–14	29	21
15–19	32	23
20–24	23	17
25–29	15	11
30–34	3	2
35–30	8	6
>40	3	2
Locality		
Jenin	36	26
Nablus	72	52
Tulkarem	31	22
Marital status		
Single	133	96
Married	6	4

**Table 2:** General characteristic and laboratory profile of the HCV-positive thalassemia cases.

Sample number	Sex	Age (years)	Thalassemia care unit	AST (U/L)	ALT (U/L)	HBV
39N	Female	14	Nablus	32	18.5	Negative
58N	Male	20	Nablus	57	49	Negative
64N	Male	13	Nablus	31	50	Negative
71N	Female	4	Nablus	39	23	Negative
13T	Female	22	Tulkarem	15	6	Positive
16T	Male	27	Tulkarem	110	115	Negative
19T	Female	14	Tulkarem	28	18	Negative
20T	Male	16	Tulkarem	38	15	Negative
23T	Female	16	Tulkarem	19	22	Negative
26T	Female	33	Tulkarem	31	25	Negative
33T	Male	23	Tulkarem	20	19	Negative
36T	Male	24	Tulkarem	40	23	Negative
40T	Female	26	Tulkarem	20	25	Negative
43T	Female	12	Tulkarem	47	39	Negative

**Table 3:** Clinical and laboratory parameters of the study group.

Parameter	Mean ± SD
Hb (g/dl)	7.9 ± 1.04
MCV (fl)	75.05 ± 10.82
AST (U/L)	55.4 ± 28.7
ALT (U/L)	46.2 ± 29.4
ALP (U/ml)	334.7 ± 201.5
Anti-HBs (U/ml)	271.8 ± 390.9

	*N (%)*

Anti-HBs ≤ 10 IU/ml	40 (28.8)
Anti-HBc +ve	25 (19)
Total HCV +ve	14 (10)
Anti-HCV +ve	7 (5)
Anti-HCV +ve and HCV-RNA +ve	4 (2.9)
Anti-HCV +ve and HCV-RNA −ve	3 (2.1)
Anti-HCV −ve and HCV-RNA +ve	7 (5.3)
Anti-HCV −ve and HCV-RNA −ve	126 (90)
History of jaundice	44 (32)
Dental procedure	71 (52)
Splenectomy	59 (82)

Hb: hemoglogin; MCV: mean corpuscular volume; AST: aspartate aminotransferase; ALT: alanine aminotransferase; ALP: alkaline phosphatase.

**Table 4:** Clinical and laboratory parameters of HCV patients and non-HCV patients.

	HCV cases	Non-HCV cases	*P* value
Hb (g/dl)			
Normal	12	116	0.19
Low	2	6	
Total	14	122	
Age			
>12	13	88	0.06
≤12	1	37	
Total	14	125	
AST (U/L)			
High	1	43	0.0056
Normal	13	55	
Total	14	98	
ALT (U/L)			
High	1	26	0.09
Normal	13	72	
Total	14	98	
ALP (U/ml)			
Normal	10	74	0.44
High	3	16	
Total	13	90	
Anti-HBc			
Positive	5	20	0.07
Negative	9	105	
Total	14	125	
History of jaundice			
Yes	8	36	0.034
No	6	89	
Total	14	125	

## Data Availability

The data used to support the findings of this study are included within the article.
